# Supplementary material for: Live imaging of heart tube development in mouse reveals alternating phases of cardiac differentiation and morphogenesis
Source: eLife. 2017 Dec 5;6:e30668. doi: 10.7554/eLife.30668 (PMC5731822; doi:10.7554/eLife.30668)
Supplement: Peer review history

# Decision letter

**DOI:** 10.7554/eLife.30668.057

**Affiliations:** Victor Chang Cardiac Research InstituteAustralia

In the interests of transparency, eLife includes the editorial decision letter and accompanying author responses. A lightly edited version of the letter sent to the authors after peer review is shown, indicating the most substantive concerns; minor comments are not usually included.

Thank you for submitting your article "Live imaging of heart tube development in mouse reveals alternating phases of cardiac differentiation and morphogenesis" for consideration by *eLife*. Your article has been favorably evaluated by Marianne Bronner (Senior Editor) and three reviewers, one of whom is a member of our Board of Reviewing Editors. The following individual involved in review of your submission has agreed to reveal their identity: Isabelle Migeotte (Reviewer #2).

The reviewers have discussed the reviews with one another and the Reviewing Editor has drafted this decision to help you prepare a revised submission.

Summary:

This is an elegant paper that tracks in real time the movements and differentiation status of cells contributing to the heart, specifically discriminating between the behaviours of the first and second heart field. The study used fluorescent reporters and confocal microscopy to obtain high resolution 3 and 4D images of early mouse hearts, including highly informative time-lapse movies of heart tube formation in wildtype embryos. It brings together many disparate and mostly 2D findings into a more coherent concept and provides a way to visualise the formation of what is called the transverse heart tube. The authors careful observations make a number of new points, including defining distinct stages of heart tube morphogenesis based on detailed 3D reconstructions that will be extremely useful for staging embryos and defining gene expression and function. The most significant finding is the discovery of a differentiation hiatus during heart tube formation that corresponds to a phase of heart tube morphogenesis separating first and second heart progenitor cell differentiation. This pause also covers the onset of cardiac function. This is an important observation that further highlights the distinction between the early and late differentiating populations of cardiac progenitor cells. Although the authors did not investigate how this pause might be regulated, for example by studying heart tube formation in mutant embryos, their approach could be adapted for such analysis in the future, with the challenge of dealing with complex genetic crosses. The transition between progenitor and differentiation has been observed as an increase in the levels of Nkx2-5-GFP and cTnT. Importantly, this is not continuous and differentiation of FHF and SHF progenitors is separated by a time window of ~5-7 hours. This point has some considerable implications for conceptualising this transition in light of the various findings to date and the difficult issues surrounding the use of Cre reagents and Cre reporters of varying efficacy upon which the concept of FHF and SHF are in part built. How the situation in mouse relates to that in Zebrafish will remain unclear until more careful studies allow a more direct comparison between the two species. To our knowledge, live Imaging of mouse heart morphogenesis has never been performed for such long period with such high degree of cellular resolution and quantitative analysis of individual cells fate. The data nicely complement previous studies showing temporal waves of precursors at gastrulation corresponding to the FHF and SHF, and provides a credible explanation for the long period between the exit of those populations with different profiles from the streak, and their allocation to the proper anatomical position. The data is of high quality, and the health of the embryos is remarkable after quite a long imaging.

Essential revisions:

1) The data suggesting that FHF and SHF differentiation and morphogenetic behaviours are set within the cardiac crescent, does not formally address whether there are meaningful functional differences in lineage state between these populations. Geographical issues rather than lineage issues may determine the timing of differentiation and morphogenesis. This should be made very clear in the Discussion.

2) Subsection “Imaging the Isl1-expressing cell lineage confirms absence of cardiac progenitor differentiation during early heart tube morphogenesis”, first paragraph. With respect to the activation of the Isl1Cre x reporter, I think it is the efficacy of the Cre not the frequency of activation that is the issue. Please clarify.

3) The reviewers disagree with the statement in the second paragraph of the Discussion that the contribution of the Isl1+ cells/SHF cells to the dorsal part of the primary HT will define these as non-chamber myocardium. This is not shown and lineage tracing by Christoffels with Tbx-Cre reagents show that non-working myocardium of the AVC and contribute significantly to chamber myocardium. Furthermore, it follows that the mosaic contribution of the SHF to the inflow/atrial region would also have to remain non-chamber myocardium. This is not shown and previous lineage tracing with Isl1 Cre and other reagents do not show exclusion form the atrial appendages (working myocardium). Please qualify appropriately.

4) Technical information about the time period between frames would be useful.

5) The Cre activation of Tomato also labels endoderm as can be seen in Figure 2A, especially in the sections. This should be commented on early in the Results, as it is later in the Isl1 section. Are endodermal cells also seen in Figure 1A? Have they been removed in the reconstructions? Please comment on the basis for the differences between the different Nkx2-5 alleles (Cre versus eGFP).

6) The authors describe the major axis at the "transversal" hemitube stage as being transversal to the AP axis. The authors should clarify whether their data suggest that this axis is realigned or simply replaced by an AP axis during the transition to the linear HT.

7) The authors are encouraged to elaborate in the Results section on what has been learnt from the dynamic imaging shown in Videos 5-8. For example, in the second paragraph of the subsection “2-photon live-imaging of early cardiac development in the mouse embryo” they state that this has provided information concerning the formation of the endocardium and lumen formation, yet they do not say what this information is. Similarly, can they expand on any new findings concerning the anterior movement of splanchnic mesoderm? Given that even the striking observation of the differentiation pause is validated using confocal imaging of fixed embryos it is important to highlight exactly what can be gained by time-lapse imaging. This clearly goes beyond concluding that increased Nkx2.5 expression reports cardiomyocyte differentiation (as stated as the conclusion of the next paragraph).

8) The Discussion is short and could be extended, in part by elaborating on some of the points mentioned above. The observation that there is a growth pause coincident with the differentiation pause also merits discussion. The coincidence of this pause with the onset of cardiac function could also be further discussed. Also, in the Discussion, the authors state that addition of the SHF to the zebrafish heart is seen as a continuum. However this is unclear, and the original work of de Pater et al. suggest SHF addition demarcates a distinct late differentiation phase, not dissimilar to the authors' new observations in the mouse.

9) Panel 4A explaining the live imaging set-up is slightly confusing. Was imaging of the second phase carried out by continuing imaging of embryos from the first phase or in separate experiments? Given the embryos can be cultured for 24 hours, were embryos imaged continuously through these two phases and if so were the results then the same for the second phase? Please clarify. It would be helpful to add a little more technical information to the first Results paragraph dealing with live imaging (subsection “2-photon live-imaging of early cardiac development in the mouse embryo”), mentioning maximal culture and imaging times and frequency of time-lapse acquisitions.

10) Can the authors provide live imaging data of the third phase during which SHF cells initiate differentiation? This would be most interesting to document in time-lapse images and would significantly reinforce their hypothesis that this phase involves recovery from the differentiation hiatus.

11) Concerning Figure 5, please clarify the significance of cells that remain columnar with low levels of GFP expression. Can the authors conclude that these are SHF cells versus endocardial cells?

12) Making conclusions about lineage commitment by following the GFP status of sister cells seems a relatively weak argument. For example, do the authors envisage that cells would move from a GFP high to low state? It would potentially be more interesting to highlight that differentiation is not associated with a cell division event.

13) It is unclear how the cells in Figure 2D and E were labeled. Please explain this more fully in the Results section.

14) How do the authors consider that some of the tomato labeled cells in Figure 6H become further apart over time? Is it possible to generate a video of these views comparable to Video 16?

# Reply: Author response

**DOI:** 10.7554/eLife.30668.058

Essential revisions:

1) The data suggesting that FHF and SHF differentiation and morphogenetic behaviours are set within the cardiac crescent, does not formally address whether there are meaningful functional differences in lineage state between these populations. Geographical issues rather than lineage issues may determine the timing of differentiation and morphogenesis. This should be made very clear in the Discussion.

We have now changed the Discussion (eighth paragraph) to emphasize that geographical position in addition to cell lineage may contribute to determine the timing of differentiation of the FHF vs. SHF cells.

2) Subsection “Imaging the Isl1-expressing cell lineage confirms absence of cardiac progenitor differentiation during early heart tube morphogenesis”, first paragraph. With respect to the activation of the Isl1Cre x reporter, I think it is the efficacy of the Cre not the frequency of activation that is the issue. Please clarify.

We have now changed this statement (Subsection “Imaging the Isl1-expressing cell lineage confirms absence of cardiac progenitor differentiation during early heart tube morphogenesis”).

3) The reviewers disagree with the statement in the second paragraph of the Discussion that the contribution of the Isl1+ cells/SHF cells to the dorsal part of the primary HT will define these as non-chamber myocardium. This is not shown and lineage tracing by Christoffels with Tbx-Cre reagents show that non-working myocardium of the AVC and contribute significantly to chamber myocardium. Furthermore, it follows that the mosaic contribution of the SHF to the inflow/atrial region would also have to remain non-chamber myocardium. This is not shown and previous lineage tracing with Isl1 Cre and other reagents do not show exclusion form the atrial appendages (working myocardium). Please qualify appropriately.

We have rephrased the paragraph to avoid misleading statements (Discussion, second paragraph).

4) Technical information about the time period between frames would be useful.

We have now added the time period between frames and total time of the videos in all video legends.

5) The Cre activation of Tomato also labels endoderm as can be seen in Figure 2A, especially in the sections. This should be commented on early in the Results, as it is later in the Isl1 section. Are endodermal cells also seen in Figure 1A? Have they been removed in the reconstructions? Please comment on the basis for the differences between the different Nkx2-5 alleles (Cre versus eGFP).

We have now commented on the contribution of tdTomato labeled cells in the endoderm at transverse heart tube stage, in Figure 2A, (subsection “3D analysis of cardiac progenitor differentiation”, first paragraph). Contribution to the endocardium was also observed (as reported by Stanley et al. 2002) and we also added this information in the text. For the purpose of the 3D reconstruction, the tdtomato labelled endodermal and endocardial cells was systematically removed manually and only the cardiac lineages FHF and SHF have been 3D-rendered. We added a supplementary figure to illustrate this clearly (Figure 2—figure supplement 2). In addition, the segmentation procedure is further explained in the Materials and methods section.

Regarding the embryo shown in Figure 1A, at the cardiac pre-differentiation stage, tdtomato signal is not detected in endodermal cells and we commented on that in the main text (subsection “3D static analysis of mouse HT formation”, first paragraph). tdtomato signal in endothelial cells (identifiable because of their typical flat morphology) can however be observed. The 3D reconstruction of the crescent shown in Figure 1A’ does not include the endoderm but does include endocardial cells. We added a supplementary figure to clearly illustrate this point (Figure 1—figure supplement 2).

We now comment on the basis for differences in expression patterns between the different Nkx2.5 alleles (subsection “3D analysis of cardiac progenitor differentiation”, third paragraph). While the Nkx2.5eGFP line is a 2.1kb enhancer line previously described as directing specific expression in the cardiac tissues (Lien. et al. 1999), the Nkx2.5cre line is a knock-in line driving cre expression in broader domains including the pharyngeal endoderm (Stanley et al. 2002).

6) The authors describe the major axis at the "transversal" hemitube stage as being transversal to the AP axis. The authors should clarify whether their data suggest that this axis is realigned or simply replaced by an AP axis during the transition to the linear HT.

Answering this question is not a simple task. We do see cells moving apart from each other over time at specific positions of the cc and now we mention this in the manuscript (subsection “Cardiomyocyte differentiation is not detected during heart tube morphogenesis”, first paragraph). These observations suggest highly anisotropic local behaviors involved in this this transition. Altogether, our movies do suggest that the transverse axis does not simply realign along the AP axis during the transition from transverse HT to the more spherical open HT (see Video 14), however the fragmentary nature of the data generated precludes conclusions at this point. We consider that we would need extended quantitative cell track analyses at higher density and registering 4D data between different experiments to fully understand how cells reorganize during this transition. This is ongoing work in the laboratory that will not be resolved in a short time and we consider it to be out of the scope of the manuscript.

7) The authors are encouraged to elaborate in the Results section on what has been learnt from the dynamic imaging shown in Videos 5-8. For example, in the second paragraph of the subsection “2-photon live-imaging of early cardiac development in the mouse embryo” they state that this has provided information concerning the formation of the endocardium and lumen formation, yet they do not say what this information is. Similarly, can they expand on any new findings concerning the anterior movement of splanchnic mesoderm? Given that even the striking observation of the differentiation pause is validated using confocal imaging of fixed embryos it is important to highlight exactly what can be gained by time-lapse imaging. This clearly goes beyond concluding that increased Nkx2.5 expression reports cardiomyocyte differentiation (as stated as the conclusion of the next paragraph).

We kindly disagree with the idea that live analysis might not be so relevant for the conclusions in this paper. We used fixed specimens of the Islet1Cre line for validation of our live observations but this mouse line has been around in many labs for many years and these conclusions were never inspired until live imaging has been achieved. Indeed, this highlights the need for live analysis, as it suggests we may be missing many relevant aspects of cardiac development by looking just at fixed embryos.

In our initial manuscript we did not want to enter fully into the relevance of splanchnic mesoderm migration (which is not just toward anterior but has an important medial directionality) as we felt it would not be completely understood until analyzed in detail at single cell resolution (see answer to point 6), however, upon the comment of the reviewer, we realize this aspect is an essential part of this manuscript and actually nicely provides a functional hypothesis for our observations. We have now added a new Results section focusing on morphogenesis in which we address the formation of the endocardial lumen (Figure 4F’, subsection “2-photon live-imaging of early cardiac development in the mouse embryo reveals morphogenetic events leading to HT formation”, second paragraph), the migration of the splanchnic mesoderm (Figure 4F and Figure 4—figure supplement 2, see the fourth paragraph of the aforementioned subsection and Video 9, subsection “Cardiomyocyte differentiation is not detected during heart tube morphogenesis”) and the changes in cell shape occurring during cardiomyocyte differentiation (“2-photon live-imaging of early cardiac development in the mouse embryo reveals morphogenetic events leading to HT formation”). The migration of the splanchnic mesoderm is in fact essential to understand HT formation and provides a plausible functional explanation for the existence of a pause in differentiation during HT formation. A new paragraph in the Discussion explains the functional relevance of these findings and the proposed hypothesis (Discussion, eighth paragraph.

8) The Discussion is short and could be extended, in part by elaborating on some of the points mentioned above. The observation that there is a growth pause coincident with the differentiation pause also merits discussion. The coincidence of this pause with the onset of cardiac function could also be further discussed. Also, in the Discussion, the authors state that addition of the SHF to the zebrafish heart is seen as a continuum. However this is unclear, and the original work of de Pater et al. suggest SHF addition demarcates a distinct late differentiation phase, not dissimilar to the authors' new observations in the mouse.

We thank the reviewers for these suggestions and we have extended our Discussion accordingly. We have now discussed the growth pause coincident with the differentiation pause and the coincidence of this pause with the onset of cardiac function (Discussion, fifth paragraph). We also changed our statement relative to the zebrafish heart since we cannot exclude the existence of a differentiation pause in this organism (Discussion, seventh paragraph).

9) Panel 4A explaining the live imaging set-up is slightly confusing. Was imaging of the second phase carried out by continuing imaging of embryos from the first phase or in separate experiments? Given the embryos can be cultured for 24 hours, were embryos imaged continuously through these two phases and if so were the results then the same for the second phase? Please clarify. It would be helpful to add a little more technical information to the first Results paragraph dealing with live imaging (subsection “2-photon live-imaging of early cardiac development in the mouse embryo”), mentioning maximal culture and imaging times and frequency of time-lapse acquisitions.

We have extended the technical information about the videos in the Results section. Also, we now provide information on the total imaging time and frequency of time-lapse acquisition in video legends (see also answer to point 4). The maximum culture time and imaging time was 24 hours (in 3 occasions) and we now also mention this in the manuscript (subsection “2-photon live-imaging of early cardiac development in the mouse embryo reveals 204 morphogenetic events leading to HT formation”, first paragraph). Although the embryos developed well for 24 hours, the chances that the region of interest moves out of focus increases with time, making those long acquisitions very challenging. Typical acquisition times varied instead between 3 to 13 hours (see the information in movie legends) and some embryos were indeed continuously imaged throughout the two phases (see for example videos 5–6). Some individual cells could also be tracked throughout the two phases. We have now added an example in the revised manuscript (Figure 6—figure supplement 1).

10) Can the authors provide live imaging data of the third phase during which SHF cells initiate differentiation? This would be most interesting to document in time-lapse images and would significantly reinforce their hypothesis that this phase involves recovery from the differentiation hiatus.

We now provide evidence for the reinitiation of differentiation during the third phase by live imaging at both the arterial and venous poles. In similarity to our observations during the first phase, using the Nkx2.5eGFP reporter line, we were able to track cells at the border between the FHF and SHF at the venous pole which, at this stage is rather externally exposed and easier to image. We started the movies during the second phase, when differentiation is not detected, and extended recordings into the third phase, when differentiation is expected to restart. We tracked cells showing initially a low and stable GFP level throughout the second phase, and found few cells increasing their GFP expression at later stages corresponding to the start of the third phase. These data have been included in Figure 8—figure supplement 2.

During the third phase, it remains however a challenge to track SHF cells differentiating at the arterial pole by detection of an increase in the Nkx2.5eGFP signal, because those cells are located deep inside the embryo and the fluorescent signal is consequently poorly detected and remains faint over time (as explained in the first paragraph of the Discussion). We have replaced the Figure 8—figure supplement 1 to make the point about this limitation. In contrast, the Rosa26Rtdtomato reporter can be detected in cells of the arterial pole by live imaging during the third phase. In the revised version of the manuscript, we have therefore added one additional live imaging analysis from an Isl1cre/R26tdtomato embryo. While no differentiation of SHF cells during the second phase could be detected (as shown in Figure 8A and C and Video 16–17), the recordings during the third phase show that SHF tdtomato positive cells initiate incorporation to the arterial pole after the open HT has formed, as the RV becomes visible (included in Figure 9C). This is confirmed in 3D reconstructions based on fixed and optically cleared embryos of a similar stage. These data confirm by live imaging that cells at the border between FHF and SHF remain undifferentiated during HT morphogenesis and resume differentiation at the end of this phase.

11) Concerning Figure 5, please clarify the significance of cells that remain columnar with low levels of GFP expression. Can the authors conclude that these are SHF cells versus endocardial cells?

Yes, those cells that remain columnar with low level of GPF expression are SHF cells and not endocardial cells. Whole mount immunostainings against CD31, an endocardial marker, shows that endocardial cells are absent from the splanchnic mesoderm, where SHF cells reside. We added a supplementary figure to show this (Figure 2—figure supplement 2). In addition, endocardial cells show typical elongated cell shapes which makes them easily identifiable in live analysis, and they are found in the cardiac crescent/heart tube lumen but not in the splanchnic mesoderm, where SHF cell reside (Figure 4F’’ and Figure 5—figure supplement 2). We have now clarified this point in the text (subsection “Live tracking of Cardiomyocyte differentiation in individual cells reveals cardiac 262 crescent differentiation dynamics”, third paragraph). Note that eGFP signal in the Nkx2.5eGFP enhancer line can be detected in the endocardium but also, albeit faintly, in some endodermal cells.

12) Making conclusions about lineage commitment by following the GFP status of sister cells seems a relatively weak argument. For example, do the authors envisage that cells would move from a GFP high to low state? It would potentially be more interesting to highlight that differentiation is not associated with a cell division event.

Yes, indeed our tracking is short-term and therefore we would not be able to tell the fate of the cells beyond the observation period if we would solely rely on our observations. However, we show that high GFP levels correlate with expression of cTnnT and long-term experiments using cTnnTcre lineage tracing indeed show that all cells expressing cTnnT are exclusively fated to the cardiomyocyte lineage (Jiao et al. 2003). We therefore think it is reasonable to assume that we have effectively traced the fate of the cells at the cardiac crescent stage by this assay. Obviously, this observation is of limited interest, provided the previous report of FHF/SHF segregation around gastrulation (Lescroart et al. 2014; Devine et al., 2014) and the restricted mixing of cardiac precursor cells (see Figure 5—figure supplement 3 but also past clonal analysis showing non-dispersion of clonally related cells in the heart, Meilhac et al. 2003), as commented in our response to point 1). Nonetheless we think there are not many examples of tracking cell division and differentiation events at single-cell resolution in vertebrates and certainly none in mammalian heart development, so we think this is an important demonstration of the potentiality of the new approach.

13) It is unclear how the cells in Figure 2D and E were labeled. Please explain this more fully in the Results section.

We have now explained in the Materials and methods section how single cells were labelled in Figure 2D, E (subsection “Cell labeling, 3D tracking and GFP Intensity Measurement”). We have also indicated the genotype of the mouse used in the Figure 2D.

14) How do the authors consider that some of the tomato labeled cells in Figure 6H become further apart over time? Is it possible to generate a video of these views comparable to Video 16?

We have now provided the video related to Figure 6H (Video 9) and it shows SHF cells indeed moving apart from each other over time (distance between the green arrows increase from 61 to 83um over 3 hours 12mn). Cell growth and/or proliferation of cells located between the labeled cells could explain why cells are moving apart. Cells located in the splanchnic mesoderm are indeed proliferative (de Boer et al. 2012). In addition, more complex cell behaviors are plausible, like intercalation of cells as part of 3D tissue remodeling. At this stage of our analysis, we have not been able to comprehensively describe these phenomena. More detailed quantitative analysis of the 3D+t cell tracking is being performed at the moment but this is a whole and challenging project out of the scope of this manuscript.
